# Effects of forward mask duration variability on the temporal dynamics of brief facial expression categorization

**DOI:** 10.1177/20416695231162580

**Published:** 2023-03-20

**Authors:** Justin A. Chamberland, Charles A. Collin

**Affiliations:** 6363 University of Ottawa, Canada

**Keywords:** emotions, facial expressions, stimulus duration, thresholds, masking

## Abstract

The Japanese and Caucasian Brief Affect Recognition Task (JACBART) has been proposed as a standardized method for measuring people's ability to accurately categorize briefly presented images of facial expressions. However, the factors that impact performance in this task are not entirely understood. The current study sought to explore the role of the forward mask's duration (i.e., fixed vs. variable) in brief affect categorization across expressions of the six basic emotions (i.e., anger, disgust, fear, happiness, sadness, and surprise) and three presentation times (i.e., 17, 67, and 500 ms). Current findings do not demonstrate evidence that a variable duration forward mask negatively impacts brief affect categorization. However, efficiency and necessity thresholds were observed to vary across the expressions of emotion. Further exploration of the temporal dynamics of facial affect categorization will therefore require a consideration of these differences.

Individuals have an inherent faculty to voluntarily control the facial expressions they portray to others, which enables them to either simulate an emotion they do not feel, minimize or neutralize an emotion they do feel, or hide their true emotion with the simulation of another (Ekman, 1985/2009; Ekman & Friesen, 1969; Zuckerman et al., 1981). The portrayal of voluntary facial expressions can, for obvious reasons, be advantageous for the expresser when they intend to manipulate others (Zuckerman et al., 1981). However, in line with Darwin's (1872) Inhibition Hypothesis, this expression control is not absolute and involuntary leakage can unintentionally result in brief demonstrations—appearing for < 0.5 s—of an emotion the individual is trying to conceal (Ekman, 1985/2009; Ekman & Friesen, 1974; Haggard & Isaacs, 1966; Yan et al., 2013). These brief emotional leakages—often termed *micro-expressions*—are suggested to differ from typical involuntary expressions, which are otherwise known as *macro-expressions* and portrayed for between 0.5 and 4 s (Ekman, 2003; Hess & Kleck, 1990).

Although empirical data concerning the production of micro-expressions are still limited due to their nature (see Porter & ten Brinke, 2008 or Yan et al., 2013), there is a great deal of research exploring their categorization (e.g., Calvo & Lundqvist, 2008; Ekman & Friesen, 1974; Esteves & Öhman, 1993; Marsh et al., 2010; Matsumoto & Hwang, 2011; Matsumoto et al., 2000, 2014; Milders et al., 2008; Porter & ten Brinke, 2010; Russell et al., 2006, 2008; Shen et al., 2012). Observers are predictably found to have increased difficulty accurately recognizing these brief expressions—as compared to full-duration macro-expressions. Indeed, longer display times were found to benefit brief affect categorization (Calvo & Lundqvist, 2008; Kirouac & Doré, 1984).

There is, however, evidence (e.g., Marsh et al., 2010, Matsumoto & Hwang, 2011, Matsumoto et al., 2014, Porter & ten Brinke, 2010, Russell et al., 2006, 2008) that individuals are capable of improving their ability to efficiently recognize these brief expressions through training, which has important implications for high-stakes situations where accurate emotion recognition could save lives (e.g., see Matsumoto et al., 2014) or enhance clinical interventions (e.g., Marsh et al., 2010; Russell et al., 2006, 2008). As a result, although outside the scope of the current study, training programs have been developed to purportedly increase a person's micro-expression recognition abilities—examples can be accessed through the Paul Ekman Group (https://www.paulekman.com/micro-expressions-training-tools/) or Matsumoto's Humintell (https://www.humintell.com/products-2/).

These training programs are of particular relevance to the current study because the Japanese and Caucasian Brief Affect Recognition Task (JACBART) was developed as a standardized measure of brief affect recognition pre- and post-training, as an assessment of training effectiveness. The JACBART proposes that a target facial expression of emotion should be presented for a brief amount of time between two neutral expressions of that target (i.e., a neutral expression of the same individual presenting the target expression is used as both the forward and backward masks). The observer is then typically asked to select which emotion the target expression portrayed (e.g., Hurley, 2012; Hurley et al., 2014; Matsumoto & Hwang, 2011; Matsumoto et al., 2000).

The introduction of the neutral expression after the target expression (i.e., a backward mask) with the JACBART—as compared to the Brief Affect Recognition Test (BART; see Ekman & Friesen, 1974)—was thought to serve an important function beyond simply increasing the measure's ecological validity. More specifically, it was thought this approach would reduce the likelihood that a brief target image would leave a residual afterimage in the early stages of the visual system, which could consequently facilitate post-offset processing of the target and lead to increased recognition rates (e.g., Bacon-Macé et al., 2005; Shen et al., 2012). Indeed, the duration (Esteves & Öhman, 1993; Macknik & Livingstone, 1998) and type (Loffler et al., 2005; Milders et al., 2008; Zhang et al., 2014) of backward mask have been observed to impact brief affect recognition thresholds. More specifically, a backward mask of at least 50 ms is suggested to significantly lower recognition rates (Esteves & Öhman, 1993; Macknik & Livingstone, 1998), and a neutral-face backward mask was found to add increased affect recognition difficulty as compared to a dynamic checkerboard backward mask (Milders et al., 2008; see, also, Loffler et al., 2005).

Despite the many studies demonstrating the impact of a backward mask on brief affect recognition, there is little research exploring the role of the forward mask (Esteves & Öhman, 1993; Macknik & Livingstone, 1998). Indeed, the JACBART commonly displays a forward mask for a fixed duration (e.g., Matsumoto & Hwang, 2011; Shen et al., 2012; Zhang et al., 2014) or a duration that varies systematically to obtain a fixed duration sum across the target and mask durations (e.g., Hurley, 2012; Hurley et al., 2014; Matsumoto et al., 2000). Furthermore, even with the latter approach, the forward mask varied by at most 65 ms within an experiment.

This presents a potential limitation for the JACBART because individuals have demonstrated an ability to devote their attention to specific timespans. For instance, primes with temporal information have been found to benefit target recognition, when compared to primes without temporal meaning (Coull & Nobre, 1998). Consequently, a fixed forward mask duration could facilitate emotion recognition and impact recognition thresholds for the JACBART by allowing participants to use the forward mask onset as a prime to predict the target onset.

The current study was therefore designed to explore the impact of forward mask duration variability on emotion categorization performances with brief expressions of emotion in a JACBART paradigm. The current study presented target expressions for either 17, 67, or 500 ms (i.e., 1, 4, or 30 frames on a 60 Hz display monitor)—as 17 ms was the shortest time for which one can present a visual stimulus on a standard 60 Hz monitor, 67 ms is the shortest time typically employed with the JACBART (e.g., Matsumoto et al., 2000), and 500 ms is thought to yield performances equivalent to unlimited viewing (Calvo & Lundqvist, 2008) and represents the threshold between micro-expressions and macro-expressions. The version of the JACBART employed by Matsumoto and Hwang (2011) was modified such that the stimuli appeared either after a *fixed duration* (1,000 ms) or *variable duration* (750–1,250 ms) forward mask. By then asking participants to correctly categorize the six basic emotions (anger, disgust, fear, happiness, sadness, and surprise) with the three target durations, the current study sought to explore forward mask variability's relationship with the *necessity threshold—*the minimum presentation time needed to recognize facial expressions at a rate significantly above chance—and the *efficiency threshold—*defined as the minimum presentation time required for a brief facial expression to be recognized at a rate statistically equivalent to a macro-expression (i.e., a duration of 500 ms or more). It was hypothesized that the variable-duration forward mask would reduce a participant's ability to predict the onset of the target expression. This increased difficulty would be reflected through lower hit rates and greater erroneous response rates, and consequently increases the necessity and efficiency thresholds. That is, both above-chance performance and ceiling-level performance would be expected to require longer target durations.

Two additional hypotheses were also formulated with respect to these categorization thresholds. First, based on research with fixed duration pre-target fixation screens (i.e., without a forward mask; Milders et al., 2008; Pessoa et al., 2005), the necessity threshold was hypothesized to vary across the six basic emotions. More specifically, it was predicted that 17 ms would be sufficient for the recognition of angry and happy expressions at rates above chance, while 67 ms would be needed for expressions of fear. For the remaining emotions, due to a lack of information in previous studies, it was unclear whether 17 ms would be sufficient to yield above-chance performances. Second, Calvo and Lundqvist (2008) suggest 67 ms may not be sufficient for categorization performances to meet the current efficiency threshold definition with any expressions of emotion. However, despite their interaction between display time and displayed emotion, they did not consider how to display time could have varying effects across emotions—opting to, instead, collapse their data across emotion conditions. It was, therefore, hypothesized that the efficiency threshold would vary across expressions of emotion.

## Methods

### Participants

Thirty undergraduate students were recruited to participate in the current study. These individuals were randomly assigned one of two forward mask groups: *Fixed-Duration* or *Variable-Duration*. There were 13 individuals in the Fixed-Duration group (5 men, 8 women; *M*_age_ = 18.38, *SD* = 0.77 years) and 17 in the Variable-Duration group (2 men, 15 women; *M*_age_ = 19.71, *SD* = 4.25 years). All participants were recruited through an undergraduate participant pool, the Integrated System of Participation in Research (ISPR), at the University of Ottawa. Participants completed the experiment individually for course credit in the Integrated Neurocognitive and Social Psychophysiology Interdisciplinary Research Environment (INSPIRE) lab at the University of Ottawa. Although they were tested in groups of up to four individuals, each completed the task on a separate computer shielded from the others by a carrel. All participants in a given session were placed in the same forward mask group.

### Materials

E-Prime v2.3 (Psychology Software Tools, Sharpsburg, PA) was used to present a total of 216 trials on ViewSonic VT2405 monitors with a 60 Hz refresh rate and 1920 × 1080 pixel resolution. Stimuli consisted of a total of 24 target images taken from the Japanese and Caucasian Facial Expressions of Emotion (JACFEE; Matsumoto & Ekman, 1988) database. This included images of two men and two women Caucasian models each displaying facial expressions of all six basic emotions (Anger, Disgust, Fear, Happiness, Sadness, and Surprise). In addition, the neutral face images of all four models were used as their respective target expression masks. All images were cropped such that the emotionally expressive faces would align with their neutral counterparts, which served to reduce the degree of apparent motion induced by the switch between the neutral mask images and the target images.

### Procedure

**Figure 1.**
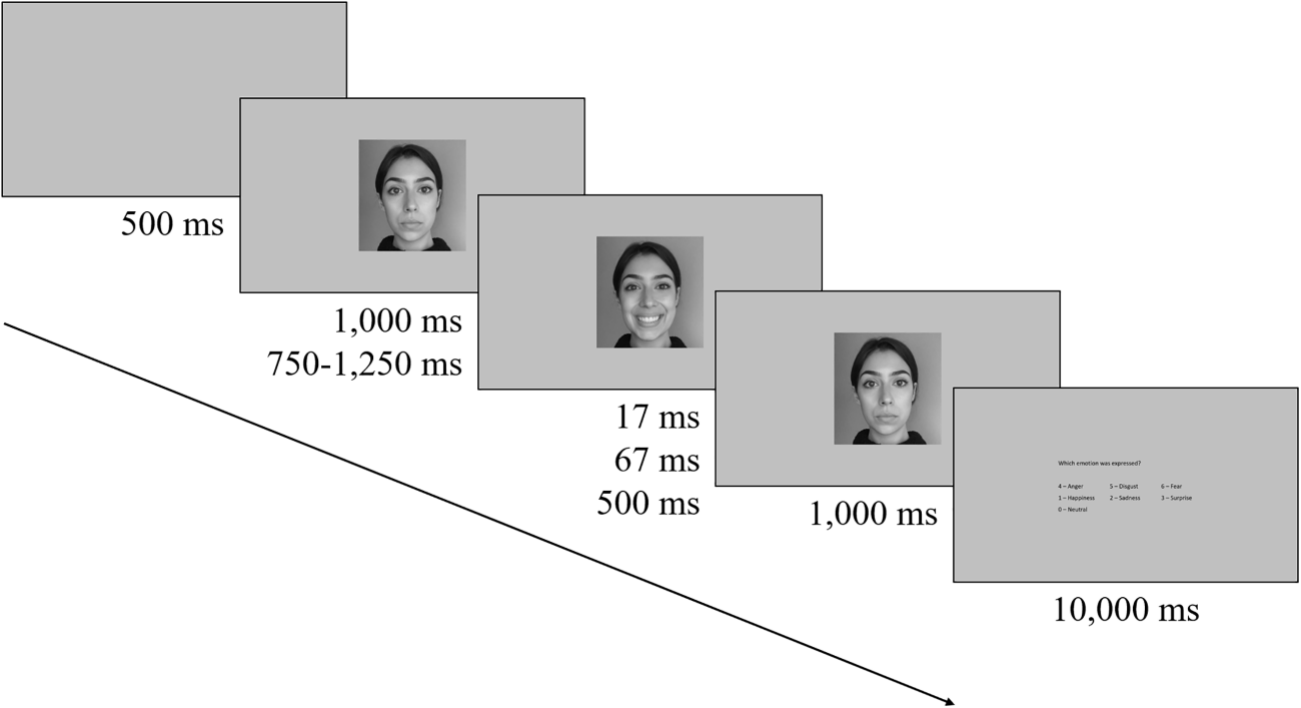
The sequence of events presented within a single trial for both forward-mask condition groups (fixed: 1,000 ms; variable: 750–1,250 ms) and the target durations (17, 67, and 500 ms).

A modified JACBART paradigm was used (Matsumoto et al., 2000; Matsumoto & Hwang, 2011), where a target expression was always presented between two neutral expressions portrayed by the same actor (see Figure 1). Each trial began with the display of a neutral facial expression for either 1,000 ms (Fixed-Duration group; see Matsumoto & Hwang, 2011) or a random duration between 750 and 1,250 ms (Variable-Duration group; with 17 ms increments). The target expression was then displayed for one of three set durations (17^1^, 67, or 500 ms) and followed by the neutral expression image for 1,000 ms. The participant was then asked to indicate which expression was displayed by selecting from the seven options: “anger,” “disgust,” “fear,” “happiness,” “sadness,” “surprise,” or “neutral.” Participants were instructed to respond as quickly and accurately as possible, and a maximum of 10 s was given to respond. Any response outside this time was deemed to be unrepresentative of the current affect categorization task (see Hurley, 2012; Hurley et al., 2014). After the participant's response, a 500 ms blank screen inter-trial interval was presented.

All stimuli were presented as 600 by 625-pixel gray-scaled images in the center of the monitor. In addition, expressions of emotion were aligned with their neutral face counterparts using the dorsum of the nose, which offered a more stable baseline across the various expressions. There was a total of 216 trials, with three target presentation durations (17, 67, and 500 ms), six target expressions of emotion (Anger, Disgust, Fear, Happiness, Sadness, and Surprise), four models (2 men and 2 women), and three repetitions. Trials were presented in random order.

### Data Analyses

Using R (version 3.6.1) software, participant responses were transformed into binary values to compute hit rates—responses that aligned with an expression's expected label—and erroneous response rates—responses that did not align with an expression's expected label. That is, when calculating hit rates, a “1” was given when a response corresponded with the image's expected emotional label, while a “0” was given when it did not. Conversely, when calculating the six erroneous response rates for each target expression, a “1” was only given to an emotional response option when that response was used and it did not correspond with the image's expected emotional label, while a “0” was used for all other cases.

To approximate the necessity threshold for each target expression, each group's hit rates were compared to chance (1/6 or 16.67%) using single-sample *t*-tests—accounting for target emotion and duration. Bonferroni adjustments were applied to *p-*values to account for the multiple comparisons. Next, to explore the efficiency threshold, hit rates were compared across conditions in a 6 (Target Emotion: Anger, Disgust, Fear, Happiness, Sadness, and Surprise) × 3 (Target Duration: 17, 67, and 500 ms) × 2 (Forward Mask: fixed-duration or variable-duration) mixed-design ANOVA. Greenhouse-Geisser corrections were applied where the assumption of sphericity was violated according to Mauchley's test, and Bonferonni adjustments were applied for all post-hoc comparisons.

Task performance was further examined through an analysis of erroneous response rates. These were analyzed in two stages: (1) chi-squared goodness-of-fit tests were used to determine if participants used any particular erroneous response more than others (expected erroneous rate = [1 − hit rate]/6) and (2) where the chi-square test showed non-uniform use of erroneous response options, all erroneous response rates were compared to chance (16.67%) in single-sample *t*-tests. Bonferroni adjustments were applied to *p*-values to account for the multiple comparisons.

## Results and Discussion

The JACBART was designed as a standardized approach for testing one's ability to recognize brief displays of emotion. This paradigm displays a target expression of emotion between presentations of two neutral expression images of the same actor. The neutral face image appearing before the target image is referred to as the forward mask, whereas the one appearing after is called the backward mask (Matsumoto et al., 2000). While there has been some work on the effects of the backward mask, there is currently little research exploring the influence of the forward mask on performance in the JACBART (Esteves & Öhman, 1993; Macknik & Livingstone, 1998). In addition, previous studies examining the categorization of briefly presented facial expression images have generally examined only a limited range of expressions (e.g., Esteves & Öhman, 1993; Milders et al., 2008; Pessoa et al., 2005) or have not analyzed differences across the expression categories (e.g., Calvo & Lundqvist, 2008).

With this in mind, the current study was designed to explore the role of the forward mask's duration, specifically the effects of changing from a fixed duration (1,000 ms) to a variable one (750–1,250 ms), on the categorization of briefly-presented facial expressions of emotion. In addition, it sought to explore the influence of the forward mask's duration on the necessity threshold—defined as the minimum target duration required to exceed chance—and efficiency threshold—defined as the point where target recognition is no longer benefitted by longer display times. To this end, using the JACBART design, three target expression durations—17, 67, and 500 ms—were used to present expressions of six basic emotions: Anger, Disgust, Fear, Happiness, Sadness, and Surprise. Mixed-design ANOVAs did not demonstrate a significant effect or interaction with the forward mask's duration (see Table 1). Conversely, a significant interaction was observed between target emotion and target duration. Results for each emotion will be discussed in turn.

**Table 1.** Mixed-design ANOVA examining hit rate differences across the forward mask conditions, target emotions, and target durations.

	*df*	*F*	$\eta_{G}^{2}$
Forward mask	1, 28	0.08	0.0007
Target emotion	3.52, 98.62	111.80***	0.56
Forward mask * target emotion	2, 56	0.94	0.01
Target duration	3.52, 98.62	277.30***	0.59
Forward mask * target duration	2, 56	1.82	0.01
Target emotion * target duration	6.03, 168.87	36.19***	0.27
Forward mask * target emotion * target duration	6.03, 168.87	1.16	0.01

^†^ < .10; *p < .05; **p < .01; ***p < .001.

### Anger Expressions

Our findings showed that with expressions of anger, in contrast to prior literature (Milders et al., 2008; Pessoa et al., 2005), 17 ms was not sufficient for expression categorization; Hit rates were not observed to exceed chance with either the fixed-duration or variable-duration forward masks (see Tables 2 and 3). Indeed, angry expressions were often erroneously recognized as “neutral” with this target duration—regardless of the forward mask condition (see Tables 2 and 3). This disparity from the findings of Milders et al. (2008) was not initially hypothesized but may be due to differences in methodology. Specifically, Milders and colleagues used a different expressor identities for the mask and target images, whereas the current design used the same expressor identity for both. As Milders et al. themselves show, the type of mask can impact performance. They observed that a neutral face mask increased the task difficult relative to a dynamic checkerboard mask. Our results can be seen as an extension of theirs, showing that when a same-identity face is used as a forward mask, it reduces performance compared to a different-identity face. This in turn means that more time is required to recognize a facial expression when a same-identity face is used as a mask.

**Table 2.** Hit rates (italicized) and erroneous response rates for the fixed-duration forward mask group (n = 13). Including chi-square goodness-of-fit tests (*df* = 5) and single-sample *t*-tests (chance = 16.67%).

Target duration	*χ* ^2^	Mean (standard error) response rates													
		Anger	**^ ^**	Disgust		Fear		Happiness		Sadness		Surprise		Neutral	
*Expressions of anger*															
17	211.54***	*0.09* (*0.03*)		0.10 (0.04)		0.01 (0.01)		0.04 (0.03)		0.05 (0.02)		0.03 (0.02)		0.67 (0.10)**	
67	45.09***	*0.48* (*0.08*)*		0.24 (0.04)		0.03 (0.02)		–		0.11 (0.03)		0.02 (0.01)		0.12 (0.05)	
500	140.47***	*0.51* (*0.09*)*		0.38 (0.09)		0.02 (0.01)		–		0.06 (0.03)		–		0.01 (0.01)	
*Expressions of disgust*															
17	87.60***	0.14 (0.05)		*0.29* (*0.06*)		0.03 (0.02)		0.08 (0.04)		0.03 (0.01)		0.03 (0.02)		0.40 (0.09)	
67	96.63***	0.31 (0.09)		*0.56* (*0.08*)**		0.03 (0.02)		0.01 (0.01)		0.01 (0.01)		0.02 (0.01)		0.05 (0.03)	
500	29.10***	0.08 (0.03)		*0.87* (*0.04*)***		0.04 (0.03)		–		–		–		–	
* Expressions of fear*															
17	109.73***	0.01 (0.01)		0.05 (0.02)		*0.13* (*0.05*)		0.03 (0.01)		0.03 (0.02)		0.28 (0.07)	^ ^	0.45 (0.11)	
67	76.90***	–		0.06 (0.03)		*0.54* (*0.06*)***		0.01 (0.01)		–		0.28 (0.05)		0.09 (0.05)	
500	21.97***	–		0.03 (0.02)		*0.86* (*0.05*)***		–		0.01 (0.01)		0.08 (0.04)		0.01 (0.01)	
* Expressions of happiness*															
17	43.01***	–		0.01 (0.01)		0.01 (0.01)		*0.84* (*0.05*)***		–		0.01 (0.01)		0.13 (0.04)	
67	4.49	0.01 (0.01)		0.01 (0.01)		–		*0.96* (*0.02*)***		–		–		0.02 (0.01)	
500	3.21	–		–		–		*0.99* (*0.01*)***		–		–		0.01 (0.01)	
* Expressions of sadness*															
17	251.46***	0.03 (0.01)		0.04 (0.03)		0.02 (0.01)		0.01 (0.01)		*0.16* (*0.06*)		0.05 (0.03)		0.68 (0.10)**	
67	35.69***	0.07 (0.03)		0.07 (0.03)		0.03 (0.02)		–		*0.62* (*0.07*)**		0.02 (0.01)		0.19 (0.06)	
500	10.35^†^	–		0.03 (0.03)		0.01 (0.01)		–		*0.96* (*0.03*)***		0.01 (0.01)		–	
*Expressions of surprise*															
17	58.29***	–		0.01 (0.01)		0.01 (0.01)		0.01 (0.01)		0.01 (0.01)		*0.82* (*0.06*)***		0.15 (0.06)	
67	7.69	–		–		0.02 (0.01)		–		–		*0.96* (*0.02*)***		0.02 (0.01)	
500	9.62^†^	–		–		0.02 (0.01)		–		–		*0.98* (*0.01*)***		–	

***p < .001; **p < .01; *p < .05; ^†^*p *< .10; one-tailed; Bonferroni adjusted.

**Table 3.** Hit rates (italicized) and erroneous response rates for the variable-duration forward mask group (n = 17). Including chi-square goodness-of-fit tests (*df* = 5) and single-sample *t*-tests (chance = 16.67%).

Target duration	*χ* ^2^	Mean (standard error) response rates													
		Anger	**^ ^**	Disgust		Fear		Happiness		Sadness		Surprise		Neutral	
*Expressions of anger*															
17	287.68***	*0.05* (*0.02*)		0.05 (0.02)		0.02 (0.01)		0.03 (0.02)		0.04 (0.02)		0.03 (0.02)		0.77 (0.08)***	
67	39.67***	*0.49* (*0.05*)***		0.23 (0.04)		0.04 (0.02)		0.00 (0.00)		0.10 (0.02)		0.01 (0.01)		0.11 (0.03)	
500	49.49***	*0.71* (*0.05*)***		0.18 (0.05)		0.02 (0.02)		–		0.05 (0.02)		0.01 (0.01)		0.01 (0.01)	
*Expressions of disgust*															
17	65.81***	0.13 (0.04)		*0.17* (*0.04*)		0.05 (0.02)		0.12 (0.03)		0.02 (0.01)		0.08 (0.03)		0.40 (0.06)*	
67	76.58***	0.28 (0.06)		*0.57* (*0.06*)***		0.04 (0.01)		0.03 (0.01)		0.00 (0.00)		0.03 (0.02)		0.04 (0.03)	
500	25.80***	0.10 (0.03)		*0.84* (*0.05*)***		0.03 (0.01)		–		0.00 (0.00)		0.01 (0.01)		0.00 (0.00)	
*Expressions of fear*															
17	148.98***	0.02 (0.01)		0.03 (0.02)		*0.10* (*0.02*)		0.05 (0.02)		0.02 (0.01)		0.25 (0.07)	^ ^	0.53 (0.07)***	
67	95.33***	0.02 (0.02)		0.01 (0.01)		*0.60* (*0.05*)***		0.03 (0.01)		–		0.29 (0.05)		0.03 (0.02)	
500	18.73**	–		0.02 (0.02)		*0.90* (*0.02*)***		–		0.01 (0.01)		0.06 (0.02)		–	
*Expressions of happiness*															
17	12.65*	0.01 (0.01)		–		0.03 (0.01)		*0.81* (*0.04*)***		0.01 (0.01)		0.06 (0.02)		0.07 (0.03)	
67	2.45	0.01 (0.01)		0.01 (0.01)		–		*0.96* (*0.02*)***		–		0.01 (0.01)		0.00 (0.00)	
500	1.23	–		0.00 (0.00)		–		*0.98* (*0.01*)***		–		0.00 (0.00)		0.00 (0.00)	
*Expressions of sadness*															
17	314.79***	0.01 (0.01)		0.05 (0.02)		–		0.00 (0.00)		*0.09* (*0.03*)		0.05 (0.03)		0.78 (0.08)***	
67	48.27***	0.04 (0.02)		0.08 (0.03)		0.03 (0.02)		–		*0.60* (*0.07*)***		0.02 (0.01)		0.22 (0.06)	
500	8.92	–		0.03 (0.03)		0.02 (0.01)		–		*0.93* (*0.04*)***		0.01 (0.01)		–	
*Expressions of surprise*															
17	41.04***	0.01 (0.01)		–		0.04 (0.02)		0.01 (0.01)		0.02 (0.01)		*0.76* (*0.05*)***		0.15 (0.04)	
67	8.63	–		–		0.03 (0.02)		0.01 (0.01)		0.00 (0.00)		*0.95* (*0.02*)***		–	
500	14.51*	–		–		0.04 (0.02)		0.00 (0.00)		0.00 (0.00)		*0.95* (*0.02*)***		–	

***p < .001; **p < .01; * < .05; ^†^*p *< .10; one-tailed; Bonferroni adjusted.

**Figure 2.**
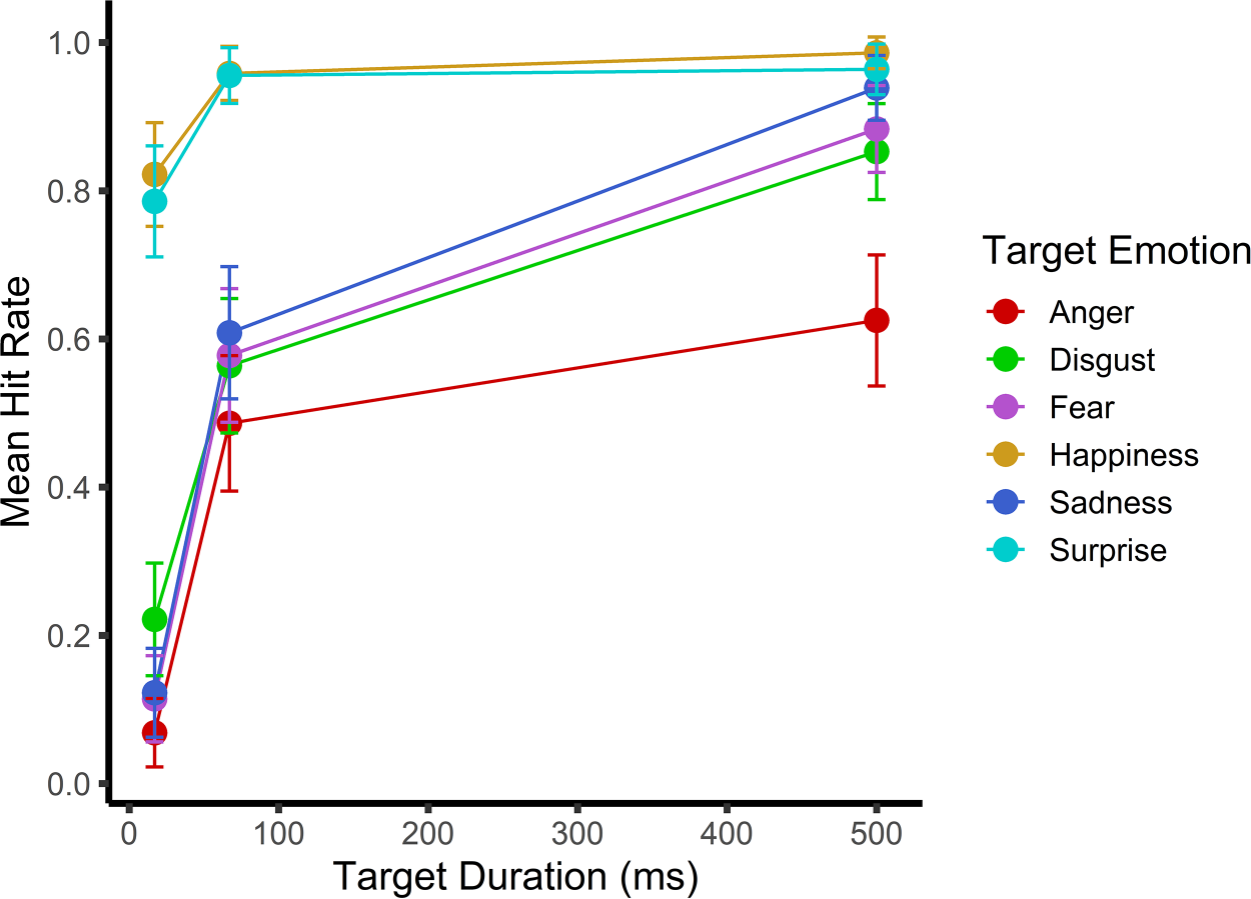
Mean hit rates for the various target emotions (anger, disgust, fear, happiness, sadness, and surprise) and durations (17, 67, and 500 ms), with combined forward-mask condition groups (fixed and variable).

Correct categorization of angry face images improved with durations longer than 17 ms (both *p* < .001). The “anger” response was used at a rate above chance with both the 67 and 500 ms displays of angry expressions regardless of the forward mask duration condition (see Tables 2 and 3). Also, there was no significant difference between the anger hit rates with these two longer target durations (*p *= .06; see Table 1 and Figure 2). Contrary to the findings of Calvo and Lundqvist (2008), this suggests anger may be efficiently recognized with as little as a 67 ms expression display time. However, inconsistent with prior literature (Gagnon et al., 2010; Gosselin et al., 1995; Jack et al., 2009), expressions of anger were not categorized with the “disgust” option at rates above chance.

### Disgust Expressions

With expressions of disgust, 17 ms was not always sufficient for correct categorization; the “disgust” response option was not used at a rate exceeding chance regardless of the forward mask condition. Indeed, participants often erroneously labeled these brief expressions as “neutral,” regardless of the forward mask condition (see Tables 2 and 3). Consistent with Calvo and Lundqvist (2008), longer display times consistently improved proper “disgust” response rates—regardless of the forward mask condition (all *p *< .001; see Table 1 and Figure 2). However, inconsistent with Calvo and Lundqvist (2008), 67 ms was sufficient for proper disgust categorization to exceed chance.

### Fear Expressions

A 17 ms presentation duration was not sufficient for participants to categorize this expression as “fear” at a rate above chance (see Tables 2 and 3). Instead, this expression often resulted in an erroneous usage of the “neutral” response when presented with a variable-duration forward mask. Hit rates were once again observed to improve with longer expression display times (all *p *< .001; see Table 1 and Figure 2), and fear expressions were properly labeled at rates above chance with both the 67 and 500 ms display times. However, inconsistent with prior literature (Chamberland et al., 2017; Gagnon et al., 2010; Gosselin et al., 1995; Gosselin & Simard, 1999; Jack et al., 2009; Roy-Charland et al., 2014, 2015), fear expressions were not confused with “surprise” after corrections for multiple comparisons.

### Happiness Expressions

Contrary to the expressions of emotion discussed above, but consistent with prior literature (Milders et al., 2008), 17 ms was sufficient for happy expressions to be accurately labeled with the “happy” response at a rate above chance. In addition, participants did not present any biases in their erroneous response options when presented with expressions of happiness (see Tables 2 and 3). Accurate “happy” response rates consistently improved as participants were presented with the happy expression for longer durations (all *p *≤ .05; see Table 1 and Figure 2)—regardless of the forward mask condition.

### Sadness Expressions

With the 17 ms display time, facial expressions of sadness were not accurately categorized (i.e., the “sadness” response option was not used) at rates above chance—regardless of the forward mask condition—and were, instead, erroneously labeled as “neutral” at rates above chance (see Tables 2 and 3). Findings show that these categorization rates improved with longer display times (all *p *< .001; see Table 1 and Figure 2), and the “sadness” label was correctly used at rates above chance with the 67 and 500 ms display durations. In addition, chi-square goodness-of-fit tests indicate there was no bias for any particular erroneous response option with the longer display time (500 ms; Tables 2 and 3).

### Surprise Expressions

As with expressions of happiness, 17 ms was sufficient for the correct categorization of surprise. Indeed, expressions of surprise were recognized at rates above chance regardless of forward mask variability condition or display time. In addition, participants did not present a bias for any particular erroneous response (see Tables 2 and 3). Despite the observed high hit rates with this expression, the “surprise” response was still accurately used at greater rates with the 67 and 500 ms display times than the 17 ms display time (both *p *< .001). However, no significant difference was observed between the two longer display times (*p *> .99; see Figure 2).

### Limitations and Future Directions

The current study was exploratory in nature with regards to the categorization thresholds and was not intended to determine precise measures of the defined thresholds. Instead, the purpose was to use these threshold measures to explore the effect of a variable-duration forward mask. Results nevertheless offer further insight and demonstrate how, in a JACBART paradigm, these thresholds can vary across facial expressions of emotion. Further research employing more presentations will be needed to determine precise stimulus duration thresholds.

Future research may want to further explore the role of a variable-duration forward mask with shorter target presentation times. However, as it is uncommon with the JACBART and biologically impossible for a micro-expression to be presented for such brief durations, said research is not entirely warranted. It should also be noted that, although the current design presented a range of forward mask variability not previously observed with the JACBART, further research is needed to determine if a wider range of variability would have a greater effect.

## Conclusion

In sum, our findings suggest the forward mask manipulation did not significantly affect categorization rates. Nevertheless, the temporal dynamics of affect categorization were observed to vary across the six basic emotions. Specifically, although 17 ms was sufficient for the categorization of expressions of happiness and surprise, a minimum of 67 ms was necessary for expressions of anger, disgust, fear, and sadness—consequently suggesting the necessity threshold could be below 17 ms with expressions of happiness and surprise, but somewhere between 17 and 67 ms with expressions of anger, disgust, fear, and sadness. Finally, 67 ms was not sufficient for efficient brief affect categorization with expressions of disgust, fear, happiness, or sadness, and the efficiency threshold is suggested to fall somewhere between 67 and 500 ms. Conversely, a relatively shorter efficiency threshold is suggested to fall between 17 and 67 ms for expressions of anger and surprise—as 67 ms was sufficient for efficient categorization rates. These data show the necessity of considering which emotional expressions one is examining when considering the speed with which individuals can recognize them. In addition, they help to bracket the durations needed to categorize a range of common expression types.

## Notes

1. Due to software limitations (see Garaizar et al., 2014), the one frame (17 ms) target stimuli were at times presented for two frames (33 ms). All 33 ms trials were removed from further analyses with the current participants.

## References

[bibr2-20416695231162580] Bacon-Macé N. Macé M. J.-M. Fabre-Thorpe M. Thorpe S. J. (2005). The time course of visual processing: Backward masking and natural scene categorisation. Vision Research, 45, 1459–1469. 15743615 10.1016/j.visres.2005.01.004

[bibr3-20416695231162580] Calvo M. G. Lundqvist D. (2008). Facial expressions of emotion (KDEF): Identification under different display-duration conditions. Behavior Research Methods, 40, 109–115. 18411533 10.3758/brm.40.1.109

[bibr4-20416695231162580] Chamberland J. Roy-Charland A. Perron M. Dickinson J. (2017). Distinction between fear and surprise: An interpretation-independent test of the perceptual-attentional limitation hypothesis. Social Neuroscience, 12, 751–768. 27767385 10.1080/17470919.2016.1251964

[bibr5-20416695231162580] Coull J. T. Nobre A. C. (1998). Where and when to pay attention: The neural systems for directing attention to spatial locations and to time intervals as revealed by both PET and fMRI. Journal of Neuroscience, 18, 7426–7435. 9736662 10.1523/JNEUROSCI.18-18-07426.1998 PMC6793260

[bibr6-20416695231162580] Darwin C. (1872). The Expression of the Emotions in Man and Animals. Oxford University Press.

[bibr8-20416695231162580] Ekman P. (1985/2009). Telling lies: Clues to deceit in the marketplace, politics, and marriage. New York, NY: W. W. Norton & Company, Inc.

[bibr9-20416695231162580] Ekman P. (2003). Darwin, deception, and facial expression. Annals of the New York Academy of Sciences, 1000, 205–221. 14766633 10.1196/annals.1280.010

[bibr11-20416695231162580] Ekman P. Friesen W. V. (1969). Nonverbal leakage and clues to deception. Psychiatry, 32, 88–106. 5779090 10.1080/00332747.1969.11023575

[bibr12-20416695231162580] Ekman P. Friesen W. V. (1974). Nonverbal behavior and psychopathology. In The psychology of depression: Contemporary theory and research (pp. xvii, 318–xvii, 318). John Wiley & Sons.

[bibr14-20416695231162580] Esteves F. Öhman A. (1993). Masking the face: Recognition of emotional facial expressions as a function of the parameters of backward masking. Scandinavian Journal of Psychology, 34, 1–18. 8322040 10.1111/j.1467-9450.1993.tb01096.x

[bibr15-20416695231162580] Gagnon M. Gosselin P. Hudon-ven der Buhs I. Larocque K. Milliard K. (2010). Children’s recognition and discrimination of fear and disgust facial expressions. Journal of Nonverbal Behavior, 34, 27–42.

[bibr16-20416695231162580] Garaizar P. Vadillo M. A. López-de-Ipiña D. Matute H. (2014). Measuring software timing errors in the presentation of visual stimuli in cognitive neuroscience experiments. PLoS One, 9, e85108. 24409318 10.1371/journal.pone.0085108 PMC3883681

[bibr17-20416695231162580] Gosselin P. Kirouac G. Doré F. Y. (1995). Components and recognition of facial expression in the communication of emotion by actors. Journal of Personality and Social Psychology, 68(1), 83–96. 7861316 10.1037//0022-3514.68.1.83

[bibr19-20416695231162580] Gosselin P. Simard J. (1999). Children’s knowledge of facial expressions of emotions: Distinguishing fear and surprise. The Journal of Genetic Psychology, 160(2), 181–193.

[bibr21-20416695231162580] Haggard E. A. Isaacs K. S. (1966). Micromomentary facial expressions as indicators of ego mechanisms in psychotherapy. In Gottschalk L. A. Auerbach A. H. (Eds.), Methods of research in psychotherapy (pp. 154–165). Springer US.

[bibr22-20416695231162580] Hess U. Kleck R. E. (1990). Differentiating emotion elicited and deliberate emotional facial expressions. European Journal of Social Psychology, 20, 369–385.

[bibr23-20416695231162580] Hurley C. M. (2012). Do you see what I see? Learning to detect micro expressions of emotion. Motivation and Emotion, 36, 371–381.

[bibr24-20416695231162580] Hurley C. M. Anker A. E. Frank M. G. Matsumoto D. Hwang H. C. (2014). Background factors predicting accuracy and improvement in micro expression recognition. Motivation and Emotion, 38, 700–714.

[bibr25-20416695231162580] Jack R. E. Blais C. Scheepers C. Schyns P. G. Caldara R. (2009). Cultural confusions show that facial expressions are not universal. Current Biology, 19(18), 1543–1548. 19682907 10.1016/j.cub.2009.07.051

[bibr26-20416695231162580] Kirouac G. Doré F. Y. (1984). Judgment of facial expressions of emotion as a function of exposure time. Perceptual and Motor Skills, 59, 147–150. 6493929 10.2466/pms.1984.59.1.147

[bibr28-20416695231162580] Loffler G. Gordon G. E. Wilkinson F. Goren D. Wilson H. R. (2005). Configural masking of faces: Evidence for high-level interactions in face perception. Vision Research, 45, 2287–2297. 15924942 10.1016/j.visres.2005.02.009

[bibr29-20416695231162580] Macknik S. L. Livingstone M. S. (1998). Neuronal correlates of visibility and invisibility in the primate visual system. Nature Neuroscience, 1, 144–149. 10195130 10.1038/393

[bibr30-20416695231162580] Marsh P. J. Green M. J. Russell T. A. McGuire J. Harris A. Coltheart M. (2010). Remediation of facial emotion recognition in schizophrenia: Functional predictors, generalizability, and durability. American Journal of Psychiatric Rehabilitation, 13, 143–170.

[bibr31-20416695231162580] Matsumoto D. Ekman P. (1988). Japanese and Caucasian Facial Expressions of Emotion (JACFEE) [Slides]. Intercultural and Emotion Research Laboratory, Department of Psychology, San Francisco State University.

[bibr32-20416695231162580] Matsumoto D. Hwang H. C. Skinner L. G. Frank M. G. (2014). Positive effects in detecting lies from training to recognize behavioral anomalies. Journal of Police and Criminal Psychology, 29, 28–35.

[bibr33-20416695231162580] Matsumoto D. Hwang H. S. (2011). Evidence for training the ability to read microexpressions of emotion. Motivation and Emotion, 35, 181–191.

[bibr34-20416695231162580] Matsumoto D. LeRoux J. Wilson-Cohn C. Raroque J. Kooken K. Ekman P. Yrizarry N. Loewinger S. Uchida H. Yee A. Amo L. Goh A. (2000). A new test to measure emotion recognition ability: Matsumoto and Ekman’s Japanese and Caucasian brief affect recognition test (JACBART). Journal of Nonverbal Behavior, 24, 179–209.

[bibr35-20416695231162580] Milders M. Sahraie A. Logan S. (2008). Minimum presentation time for masked facial expression discrimination. Cognition and Emotion, 22, 63–82.

[bibr36-20416695231162580] Pessoa L. Japee S. Ungerleider L. G. (2005). Visual awareness and the detection of fearful faces. Emotion, 5, 243–247. 15982091 10.1037/1528-3542.5.2.243

[bibr37-20416695231162580] Porter S. Brinke L. ten. (2010). The truth about lies: What works in detecting high-stakes deception? Legal and Criminological Psychology, 15, 57–75.

[bibr38-20416695231162580] Porter S. ten Brinke L. (2008). Reading between the lies: Identifying concealed and falsified emotions in universal facial expressions. Psychological Science, 19, 508–514. 18466413 10.1111/j.1467-9280.2008.02116.x

[bibr40-20416695231162580] Roy-Charland A. Perron M. Beaudry O. Eady K. (2014). Confusion of fear and surprise: A test of the perceptual-attentional limitation hypothesis with eye movement monitoring. Cognition and Emotion, 28, 1214–1222. 24460373 10.1080/02699931.2013.878687

[bibr41-20416695231162580] Roy-Charland A. Perron M. Young C. Boulard J. Chamberland J. A. (2015). The confusion of fear and surprise: A developmental study of the perceptual-attentional limitation hypothesis using eye movements. The Journal of Genetic Psychology, 176, 281–298. 26244819 10.1080/00221325.2015.1066301

[bibr42-20416695231162580] Russell T. A. Chu E. Phillips M. L. (2006). A pilot study to investigate the effectiveness of emotion recognition remediation in schizophrenia using the micro-expression training tool. British Journal of Clinical Psychology, 45(4), 579–583. 17076965 10.1348/014466505X90866

[bibr43-20416695231162580] Russell T. A. Green M. J. Simpson I. Coltheart M. (2008). Remediation of facial emotion perception in schizophrenia: Concomitant changes in visual attention. Schizophrenia Research, 103, 248–256. 18565733 10.1016/j.schres.2008.04.033

[bibr45-20416695231162580] Shen X. Wu Q. Fu X. (2012). Effects of the duration of expressions on the recognition of microexpressions. Journal of Zhejiang University Science B, 13, 221–230. 22374615 10.1631/jzus.B1100063 PMC3296074

[bibr46-20416695231162580] Yan W.-J. Wu Q. Liang J. Chen Y.-H. Fu X. (2013). How fast are the leaked facial expressions: The duration of micro-expressions. Journal of Nonverbal Behavior, 37, 217–230.

[bibr47-20416695231162580] Zhang M. Fu Q. Chen Y.-H. Fu X. (2014). Emotional context influences micro-expression recognition. PLoS One, 9(4), e95018. 24736491 10.1371/journal.pone.0095018 PMC3988169

[bibr48-20416695231162580] Zuckerman M. DePaulo B. M. Rosenthal R. (1981). Verbal and nonverbal communication of deception. In Berkowitz L. (Ed.), Advances in experimental social psychology (Vol. 14, pp. 1–59). Academic Press.

